# Patterns of neural differentiation in melanomas

**DOI:** 10.1186/1423-0127-17-87

**Published:** 2010-11-16

**Authors:** Bhanu Iyengar, Avantika V Singh

**Affiliations:** 1 Histochemistry Department, Institute of Pathology, New Delhi - 110037, India

## Abstract

**Background:**

Melanomas, highly malignant tumors arise from the melanocytes which originate as multipotent neural crest cells during neural tube genesis. The purpose of this study is to assess the pattern of neural differentiation in relation to angiogenesis in VGP melanomas using the tumor as a three dimensional system.

**Methods:**

Tumor-vascular complexes [TVC] are formed at the tumor-stroma interphase, by tumor cells ensheathing angiogenic vessels to proliferate into a mantle of 5 to 6 layers [L1 to L5] forming a perivascular mantle zone [PMZ]. The pattern of neural differentiation is assessed by immunopositivity for HMB45, GFAP, NFP and synaptophysin has been compared in: [a] the general tumor [b] tumor-vascular complexes and [c] perimantle zone [PC] on serial frozen and paraffin sections. Statistical Analysis: ANOVA: Kruskal-Wallis One Way Analysis of Variance; All Pairwise Multiple Comparison Procedures [Tukey Test].

**Results:**

The cells abutting on the basement membrane acquire GFAP positivity and extend processes. New layers of tumor cells show a transition between L2 to L3 followed by NFP and Syn positivity in L4&L5. The level of GFAP+vity in L1&L2 directly proportionate to the percentage of NFP/Syn+vity in L4&L5, on comparing pigmented PMZ with poorly pigmented PMZ. Tumor cells in the perimantle zone show high NFP [65%] and Syn [35.4%] positivity with very low GFAP [6.9%] correlating with the positivity in the outer layers.

**Discussion:**

From this study it is seen that melanoma cells revert to the embryonic pattern of differentiation, with radial glial like cells [GFAP+ve] which further differentiate into neuronal positive cells [NFP&Syn+ve] during angiogenic tumor-vascular interaction, as seen during neurogenesis, to populate the tumor substance.

## Background

Mammalian melanocytes originate as multipotent neural crest cells that detach from the neural tube to arrive at the dorsolateral surface by day 8 [1,2]. Melanomas are highly malignant tumors arising from the melanocytes, which are present primarily in the basal layer of the epidermis, but are found in various other locations such as uveal tract of the eyes, inner ear, mucous membrane, genital organs, anus and leptomeninges [3]. Cutaneous melanoma is a tumor derived from activated or genetically altered epidermal melanocytes, the result of complex interactions between genetic, constitutional, and environmental factors [4]. Malignant melanoma may arise from melanocytes in normal appearing skin, activated melanocytes of solar lentigo, or less frequently from atypical or relatively benign nevomelanocytic lesions. The incidence and mortality of cutaneous malignant melanoma has substantially increased among all Caucasian populations in the last few decades. Susceptibility to melanomas are influenced by various factors such as familial incidence, race, background, skin types and gender; constitutional factors such as age, number, size and type of pigmented nevi; accumulative and lifetime exposure to solar light [5].

The ability of melanoma cells to undergo proliferation in three dimensions is clinically known as the vertical growth phase (VGP). VGP melanoma is a highly angiogenic and proliferative lesion. Further genetic changes convert melanoma into an invasive tumor capable of three dimensional growth, increased angiogenesis, and metastasis [6,7]. The purpose of this study is to assess the pattern of neural differentiation within the tumor substance of a series of melanomas in vertical growth phase [VGP], using the tumor as a three dimensional system.

## Materials and methods

**Figure 1:**
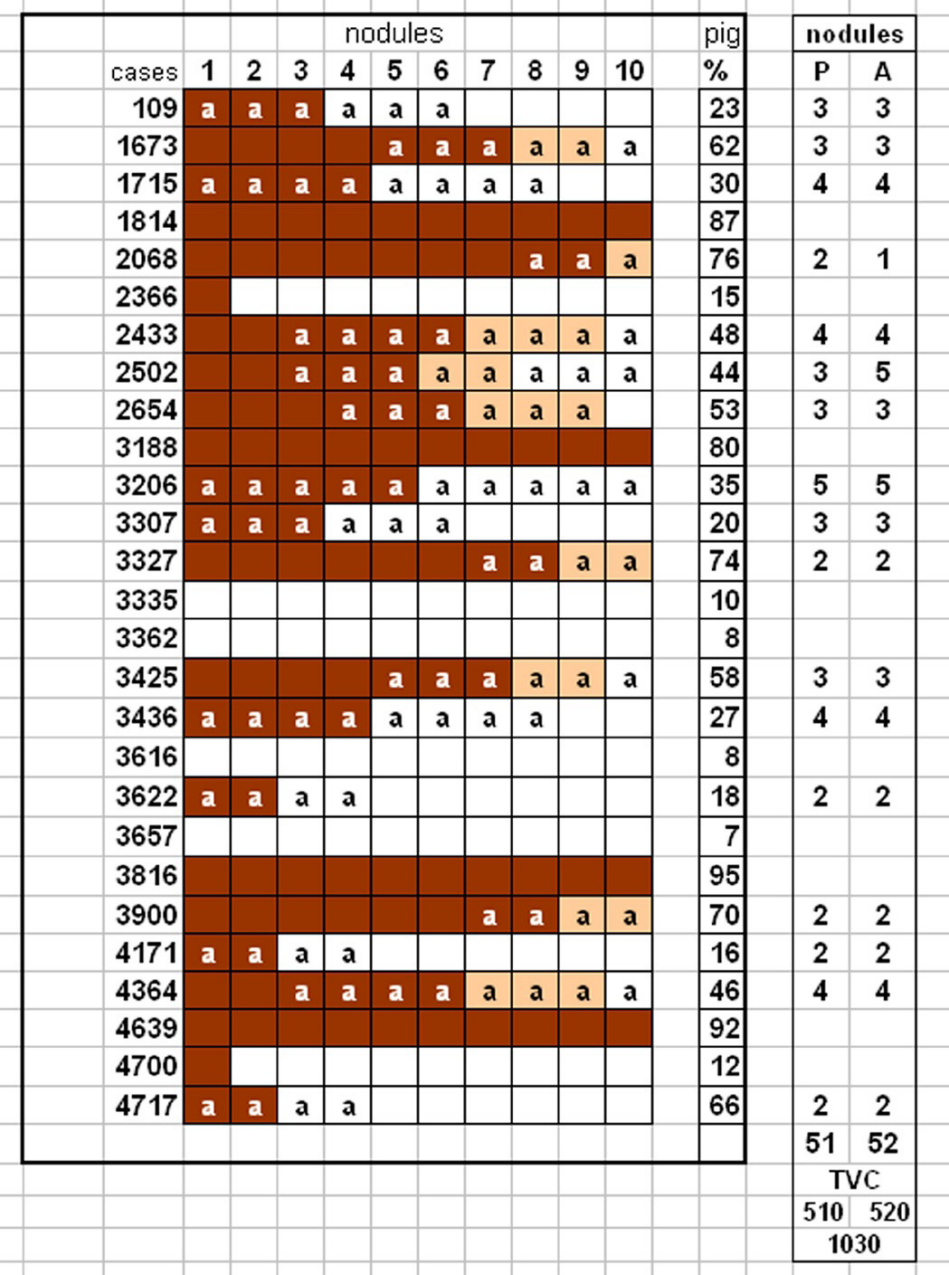
**Figure showing the pigmented vs poorly pigmented nodules arranged in a grid in order of the level of pigmentation in the tumors studied**. Nodules marked 'a' have been sectioned to study angiogenesis and tumor vascular interaction at the tumor stroma interphase.

A random sample of 27 nodular melanomas in the vertical growth phase [VGP], were received from the Cancer Surgery Unit fixed in 10% formol glutaraldehyde. The formaldehyde-glutardehyde cold fixation can be used both in frozen, paraffin sections as well as electron microscopy. 10 nodules were taken from each tumor in the in the ratio of pigmented to poorly pigmented areas in the entire tumor. As the specimen were received and sampled the blocks were arranged in a grid, according to the pigment level which varied between 7% to 95% [Figure 1].

Serial sections 5 μm thick (20-40) frozen sections and paraffin sections were cut from each block and maintained under refrigeration at 4°C. These were subjected to routine histochemistry, [HE, PAS, reticulin] [8] enzyme histochemistry [Dopa Oxidase] and immunohistochemistry using the Avidin/Biotin system [HMB45, NFP, GFAP, Synaptophysin (Syn)], [BioGenex] [9-11]. As negative control all slides included a serial section stained with no mAb. The same mAb were used simultaneously against known positive sections from human skin as positive controls.

Presence of pigment; a positive DOPA reaction; and HMB-45 positivity are criteria for diagnosis. In the absence of pigment a positive dopa reaction, HMB45 positivity and the presence of premelanosomes on electron microscopy is diagnostic of poorly pigmented melanomas. These criteria form the basis of diagnosing each tumor included in this study.

### Immunohistochemistry

Neural marker positivity has been examined and compared in:

[a] the general tumor;

[b] perimantle zone [PMZ] of tumor-vascular complexes [TVC] formed during angiogenesis;

[c] perimantle cells [PC]

Marginal zone between the tumor and stroma were selected to study the tumor/vascular interaction during angiogenesis. 51 blocks are from pigmented and 52 from poorly pigmented nodules [Figure 1].

#### Vascular counts: [Figure 2]

**Figure 2:**
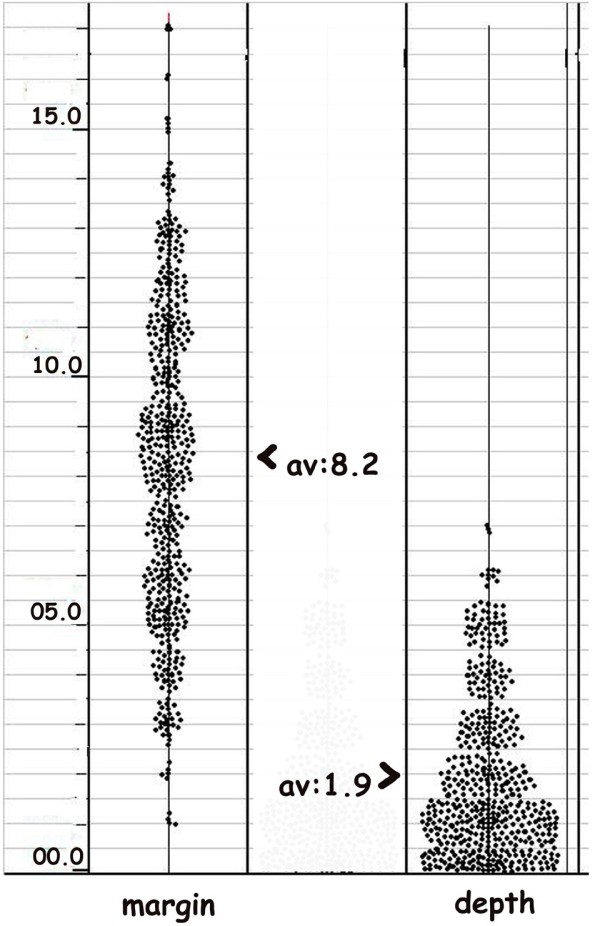
**A scatter diagram comparing the number of vessels at the margin and within the tumor**. The number of angiogenic vessels is significantly higher at the margin adjacent to the stroma.

Vascular channels are counted at the tumor margins in each of the 103 blocks to a depth of two high power fields [HPF] and at a depth of 5 to 6 HPF within the tumor in 10 HPF [1030 HPF marginal and 1030 HPF within tumor].

#### Tumor/vascular Complex [TVC]: [Figure 3 &4]

**Figure 3:**
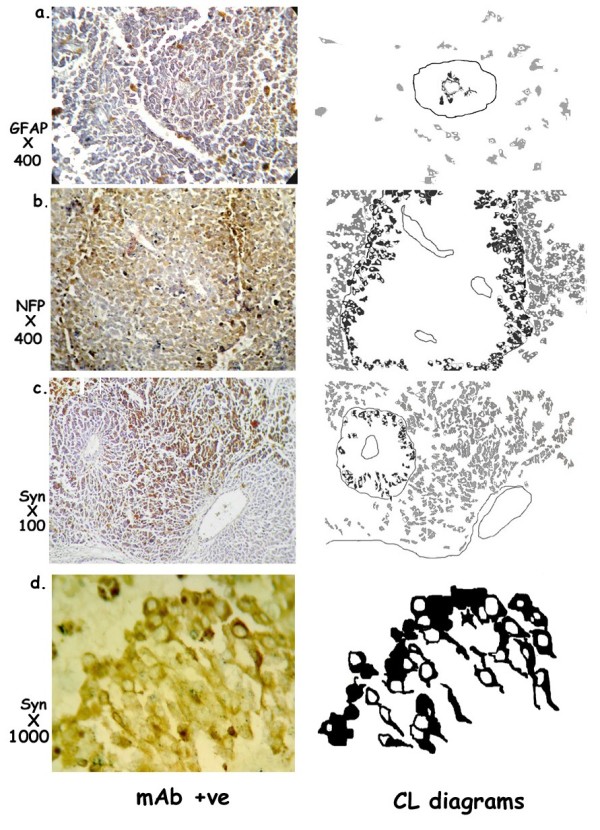
**Tumor vascular complexes showing [a] GFAP positivity in the perivascular layers 1&2 [mAbGFAPX400]; [b] NFP positivity in layers 4 & 5 [mAbNFPX400]; and [c] Syn positivity in layer 5 [mAbSynX100]**. The cells in the perimantle zone show a high percentage of NFP & Syn +ve cells and a low percentage of GFAP +ve cells. [d] Syn positive cells in L4&L5 [mAbSynX1000] showing dendritic processes resembling primitive neurons. Camera Lucida CL diagrams are shown to highlight the positive cells.

**Figure 4:**
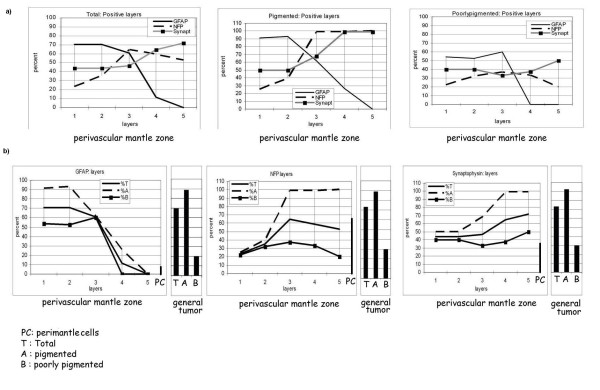
**Graph showing layer positivity [a] Comparison of immunopositivity for GFAP, NFP and Syn in total, pigmented and poorly pigmented TVC [b] GFAP, NFP & Syn +vity in the PMZ, perimantle cells and the general tumor**.

##### Perivascular mantle zone [PMZ]

The interacting tumor cells form a mantle around the angiogenic channels at the stroma/tumor interphase forming spheroidal structures. 10 random angiogenic vessels with a mantle of 5 to 6 layers of tumor cells were assessed in each serial section for the marker positivity. A total of 1030 TVC were assessed which includes 510 [A] pigmented and 520 [B] with scanty pigment. For analyses the layers around the vessel are numbered from L1 to L5 with L1 being closest to the vessel. The percentage positivity for GFAP, NFP, and Syn of each layer is represented as graphs [Figure 4], to show the marker localization in relation to the angiogenic central vessel. Specific morphological features in the different layers have been studied to correlate with the immunopositivity.

#### Perimantle cells

The percentage immunopositive cells around the mantle zone were counted to a depth of one HPF.

#### Statistical Analysis

Anova Analysis: Kruskal-Wallis One Way Analysis of Variance; and Tukey Test: All Pairwise Multiple Comparison Procedures.

## Results

### Pattern of Neural differentiation

The expression of neural markers [GFAP, NFP and Syn], by melanocytes in association with pigmentation and the tumor morphology has been examined in this section. It is observed that the general tumor areas differ from areas of angiogenesis where there is a patterned neural expression and melanocyte morphology.

#### General Tumor

There is a marked anisocytosis and anisonucleosis. Pleomorphism, increased nuclear-cytoplasmic ratio, hyperchromatin, enlarged nucleoli, abnormal mitoses and giant cells are seen. Mononucleate and multinucleate giant cells with 10-12 nuclei are also present. There is no definite pattern of neural differentiation in areas unrelated to angiogenesis.

**Total nodules **[270 nodules]: 69% [186 nodules] of the all areas studied express the three neural markers [GFAP, NFP, Syn]; 69.2% [187 nodules] of the melanomas were positive for GFAP. NFP positivity is observed in 73.1% [197 nodules], 73.1% were positive for Syn [Figure 4].

**Pgmented nodules **[135 nodules]: Pigmented nodules showed extensive positivity for the neural markers, positivity was seen in: total: 89.8% [121 nodules]; GFAP: 88.5% [120 nodules]; NFP: 88.5%[120 nodules]; Syn: 92.3% [125 nodules] [Figure 4].

**Poorly pigmented nodules **[135 nodules]: 26.7% [36 nodules] were positive for neural markers, GFAP positivity was seen in 20% [27 nodules]; NFP and Syn 30% [40 nodules] positivity was seen in the amelanotic areas [Figure 4].

### Pattern of neural differentiation in relation to Angiogenesis

The pattern of neural differentiation and cell morphology is regimented and well defined at the tumor/stroma interphase where the tumor cells interact with the neovascular angiogenic vessels. This pattern is lost within the general tumor away from the margins.

### Angiogenesis: [Figure 2]

The adjacent stromal blood vessels proliferate, to extend endothelial buds which grow towards the tumor margin. These cannelise and acquire a basement membrane at the tumor margins. The blood vessels branch extensively within the tumor substance.

Angiogenesis is significantly higher at the margins as quantified by counting the blood vessels [bv] at the margins and well within the tumor growth. On an average 8.18 bv/HPF are observed near the invasive margins and an average of 1.9 bv/HPF in the tumor. At the margins a maximum of 19 bv/HPF and a minimum of 5 bv/HPF are observed. In the areas of main tumor growth a maximum of 4 bv/HPF and a minimum of 0 bv/HPF are observed [Figure 2] Thus as there is a significant difference between angiogenic vessels at the invasive margins and within the tumor, in a rapidly growing tumor the central portions recede from the margins and are deprived of vascularisation. The tumor cells interact with the angiogenic vessels at the margins to form a mantle of 5 to 6 cell layers giving a lobular or spheroid appearance.

### Tumor vascular interaction: [Figure 3a-d]

A single layer of tumor cells surround the endothelial tubes and grow out into 5 to 6 concentric layers to form a compact spheroidal structure clearly demarcated from the surrounding tumor.

### Pattern of neural differentiation related to neovasculature: [Figure 3&4]

The pattern of differentiation in the tumor cell layers around the angiogenic vessel, is examined for neuronal markers GFAP, NFP and Syn. Quantitation and comparison has been given below.

#### Total PMZ

**GFAP**: Maximum GFAP positivity is in the layers closer to the blood vessel being 70.6% in each of the L1 and L2 with 727 of 1030 PMZ showing positivity. GFAP positivity is 61% [628/1030 PMZ] and 11.8% [122/1030 PMZ] in L3 and L4. GFAP is absent in the outermost layer i.e. L 5.

**NFP**: NFP positivity is 23.5% [242/1030 PMZ] and 35.3% [364/1030 PMZ] in the L1 and L2 respectively. Maximum NFP positivity is in the L3 (64.7%) [666/1030 PMZ] followed by L4 (59.2%) [610/1030 PMZ] and L5 (52.9%) [545/1030 PMZ]

**Syn**: Syn positivity is 44% in both L1 and L2 [453/1030 PMZ], and 46.4% in L 3 [478/1030 PMZ]. Maximum positivity is in the L4 and L5 (64.7% &72%) [666 & 742/1030 PMZ].

#### A. Pigmented PMZ [510]

**GFAP**: Highest GFAP positivity is in the L1 and L2 (91.5% & 93%) [467 & 474 of 510 PMZ]. In the outer layers of the spheroid the GFAP positivity is 62% [316/510] in L3 and 26.6% [136/510 PMZ] in L4. None of the tumor areas are positive in the L5.

**NFP**: NFP positivity is low in the inner layers being 25% [128/510 PMZ] in L1 and 39.7% [202/510 PMZ] in L2. Maximum NFP positivity (98.8%) [504/510 PMZ] is in the L3, 99.1% [505/510 PMZ] in L4 and 100% in L5, maximum positivity being in L3 to L5.

**Syn**: Syn positivity is higher in the outer layers of the PMZ as compared to the inner layers. The positivity is 50% [255/510 PMZ] in L1, L2 and 67.7% [345/510 PMZ] in L3. In the L4 and L5 the positivity increases being 98.9% [504/510 PMZ] and 99.1% [505/510 PMZ] respectively Thus peak positivity is in the L5.

#### B. Poorly pigmented PMZ [520]

**GFAP**: GFAP is expressed in the L1 and L2 where it is 54% & 52.7% [281 & 274/520 PMZ]. The positivity is 60% [312/520 PMZ] in L3 and absent beyond that in the L4 and L5.

**NFP**: The overall positivity is low as compared to pigmented spheroids. NFP positivity is seen in the L1: 22.3% [116/520 PMZ], 32% [166/520 PMZ] in L2 and 37% [192/520 PMZ] in L3, 33.3% [173/520 PMZ] in L4 and 20% [104/520 PMZ] in L5 showing maximum in L3.

**Syn**: Syn positivity is 40% [208/520 PMZ] in the L1, L2 33.3% [173/520 PMZ] in L3, 37.4% [195/520 PMZ] in L4. The positivity is 50% [260/520 PMZ] in L 5.

On Anova Analysis GFAP is significantly higher than NFP/Syn in L1&2 [(P = 0.030). F: 13.885] and significantly lower in L4&5 [(P = 0.004). F: 59.878 in L4&5]. Tukey test: All Pairwise Multiple Comparison Procedures: Comparison:P P < 0.050: GF vs. NFP:0.031 Yes; Syn vs. GF:0.004 Yes

### Perimantle Zone Cells: [Figure 4&5]

**Figure 5:**
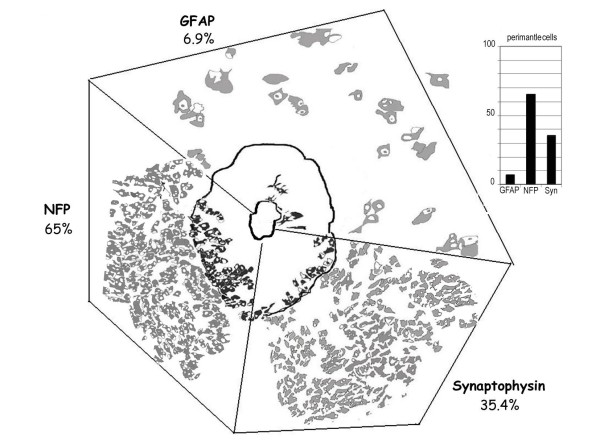
**Collage of camera lucida diagrams to compare neuronal immunopositivity in the PMZ and the perimantle zone cells [PC]**. The percentage of peripheral cells correlates with the level of marker positivity in the peripheral layers of the PMZ.

The percentage of perimantle cells [PC] vary. GFAP+ve cells form 6.9% of the PC, while NFP+ve cells form 65% and Syn+ve form 35.4% of the cells. This correlates with the immunopositivity seen in the peripheral layers of the mantle.

### Morphology

The cells in the different layers have defined morphologies unlike the general tumor. The L1&2 with GFAP positivity show dendritic cells radial glia-like cells which extend processes outward into the proliferating layers. In the outer layers L4&5 Syn positive cells extend processes inwards to resemble neuronal cells. [Figure 3d]

Occasional neovascular channels remain quiescent with a single layer of GFAP negative tumor cells outside a thin silver positive BM. There is no proliferation as seen with GFAP positive layer. At the tumor margin, the new vessels are surrounded by fibrous tissue and evolve into stromal vessels. The surrounding sheets of tumor cells unrelated to vessels, show scattered GFAP, NFP and Syn positivity but no dendricity.

## Discussion

Tumor growth and proliferation is not totally chaotic and uncontrolled as often misconstrued. This study provides an interesting aspect of the methods within the madness of malignant growth in melanomas. Melanomas provide a mass of cells as one sees in a 3D matrix. Analysis of the growth patterns would be of benefit for the study of embryonic growth patterns as well as for the study of stem cells. The patterns of neuronal differentiation have been detailed in this work including the localisation of neural markers [GFAP, NFP and Syn] by tumor cells in relation to pigmentation. There is a distinct difference between the general tumor matrix and areas of angiogenesis where there is a patterned neural expression and melanocyte morphology.

GFAP positivity identifies the radial glial multipotent astrocytic stem cells [MASC] during embryogenesis as described in several studies [12-16]. GFAP, a 50 kDa intracytoplasmic protein, constitutes the major cytoskeletal protein in astrocytes and is traditionally referred to as a specific marker for astrocytes of the CNS [13] GFAP positivity and glial differentiation is related to pigmentation and is inversely proportional to astrocytic anaplasia as is well brought out in this study [17].

Melanomas are highly angiogenic and proliferative lesions in the vertical growth phase [VGP] [18,19]. Angiogenesis is the sprouting of blood vessels from preexisting ones where endothelial buds grow out towards the tumor margins [20-24].

Reciprocal paracrine interactions between astrocytes, endothelial cells and ependymal cells have been demonstrated in recent studies. Vascular endothelial growth factor (VEGF) is released from both astrocytes and neurons eliciting a burst of mitotic angiogenesis, which is followed by the production of brain-derived neurotrophic factor (BDNF) by the stimulated microvascular cells [25-27]. In foci of concurrent angiogenesis and neurogenesis, neuronal progenitor cells are spatially associated with mitotic endothelial cells, [28-31].

From this study it is observed that the melanoma cells express characteristics of radial glia, on interaction with the endothelial tubes and further proliferate and differentiate into cells positive for neuronal markers and thus resemble MASC which give rise to neuronal differentiation in neurospheres in cultures [14,32-52].

At the tumor/stroma interphase the sprouted endothelial tubes cannelise and acquire a reticulin positive basement membrane. Initially, a single layer of tumor cells surround the vessels on the outer surface of the basement membrane. The cells abutting on the basement membrane acquire GFAP positivity and extend processes. Concentric layers of tumor cells grow out from this layer, supported by GFAP positive processes which extend outward through the layers of tumor cells towards the periphery [Figure 3a]. Where GFAP positivity is absent there is no further proliferation. As the new layers of tumor cells grow out there is a zone where all three markers are co-localized between L2 to L3 followed by NFP and Syn positivity in L4&L5.

Neurofilaments are neuron-specific intermediate filaments which can be localized by NFP positivity for neuronal differentiation [12]. They form the dynamic axonal cytoskeleton together with other axonal components such as microtubules to maintain and regulate neuronal cytoskeletal plasticity [reviewed by Kesavapany et al, 2003] [46]. During development neuroepithelial cells in the neuronal lineage lose nestin and vimentin [47] to express NF-H when the maturing cells are forming synapses [48,49]. NFP positivity is seen in differentiated ganglion cells, neoplasms of neuronal or mixed cell origin as well as neuroendocrine tumor cells. Ramirez et al [50] found rabbit choroidal melanocytes, perivascular and intervascular fibers positive for NFP.

Synaptophysin is a vesicular integral membrane protein specifically expressed in neural tissues [51]. Synaptophysin labels small synaptic like microvesicles (SLMV) present in neuroendocrine cells such as the pituitary and adrenal medulla. Synaptophysin and synaptobrevin are abundant membrane proteins of neuronal small synaptic vesicles. These vesicles characterized by synaptophysin contain considerable amounts of the biogenic amines [51,52]. Earlier studies have identified the presence of biogenic amines in melanocytes. These include catechol amines as well as indole amines [53-58].

The percentage of GFAP+vity in L1&L2 correlates with the percentage of NFP/Syn+vity in L4&L5. In the poorly pigmented PMZ the very low GFAP+vity is associated with a low NFP/Syn +vity. NFP does not increase beyond L3. This is in contrast to the pigmented PMZ where high GFAP+vity in L1/L2 is associated with a similar spike in NFP/Syn+vity in L4/L5 suggesting that the neuronal positivity results from the GFAP+vity after passing through a transitional phase. Thus in those areas where the level of differentiation is low as seen by the absence of pigment, the differentiation of the tumor cells into glial cells on interaction with the neovascular channel is low. This in turn results in low neuronal differentiation.

Immunopositivity in the immediate proximity of the PMZ in the perimantle zone reflects that of the peripheral layers of the mantle there being a very low GFAP+vity [6.9%], and high Syn [35.4%] and NFP [65%] positivity. This suggests that most GFAP +ve cells proliferate into NFP and Syn +ve cells which then populate the tumor [Figure 5].

The sequence of progression from radial glial to neuronal positive cells in the [PMZ] simulates the differentiating patterns in *invitro *neurospheres and early embryogenesis of the neural tube. The astrocyte-like stem cells have the ability to generate neurons [36-40], while newly-generated neurons can assume or revert to an astrocytic phenotype. In differentiating primary floating neurospheres neurons can shift into cells with astrocyte characteristics by transitioning through an "asteron" (neuron/astrocyte hybrid) morphotype which coexpress a variety of neuron and astrocyte proteins and genes [42].

From this data it is seen that in melanomas which are known for pleomorphism and highly variant morphology, there is an organized pattern of differentiation as the tumor spreads and vascularises. Interaction with the neovascular angiogenic channels functions as during neurogenesis. As the single interacting cell layer proliferates into a layered mantle a wave of step wise differentiation from tumor cells to glial followed by neuronal cells positive for NFP and Syn occurs.

These cells then merge with the expanding tumor cells to populate it with GFAP, NFP and Syn +ve cells which acquire the haphazard pattern seen in the general tumor substance. This mode of patterned growth is prominent in the pigmented nodules and is low in the poorly pigmented nodules and rare in the amelanotic melanomas. Thus the more differentiated the tumor the more regimented the growth pattern.

These results show that the melanoma cells have the potential for differentiating into glial as well as neuronal cells. The formation of structured PMZ during tumor cell-vascular interaction recapitulates embryogenic neurogenesis. Melanoma cells could potentially serve as neuronal stem cells, when grown as cocultures with angiogenic/endothelial cells, since in the tumor system the regimentation is confined to the PMZ, beyond which the neoplastic cells revert to a chaotic growth pattern. Although dendritic, Syn positive cells, resembling early neurons are seen in the outer layers of the PMZ [Figure 3d], *in vitro *studies are required to confirm this potential. In addition the metabolic activity of melanoma derived stem cells have to be carefully monitored.

## Competing interests

The authors declare that they have no competing interests.

## Authors' contributions

BI conceived, designed, coordinated the study as part of ongoing work on various aspects of melanocyte functions. Also did the analysis and write up of the final manuscript. AVS carried out the immunohistochemistry and the counts on the serial sections for each monoclonal antibody in the TVCs. All authors read and approved the final manuscript.
